# Effect of peroxiredoxin 1 on the regulation of trophoblast function by affecting autophagy and oxidative stress in preeclampsia

**DOI:** 10.1007/s10815-023-02820-0

**Published:** 2023-05-25

**Authors:** Meijuan Zhou, Junjun Guo, Shuxian Li, Anna Li, Zhenya Fang, Man Zhao, Meihua Zhang, Xietong Wang

**Affiliations:** 1 Key Laboratory of Birth Regulation and Control Technology of National Health Commission of China, Maternal and Child Health Care Hospital of Shandong Province Affiliated to Qingdao University, 238 Jingshi East Road, Jinan, 250014 Shandong China; 2 Department of Obstetrics and Gynecology, Shandong Provincial Hospital Affiliated to Shandong First Medical University, 324 Jingwu Street, Jinan, 250021 Shandong China

**Keywords:** Preeclampsia, PRDX1, Trophoblast, Autophagy, ROS

## Abstract

**Purpose:**

PE is a pregnancy-specific syndrome and one of the main causes of maternal, fetal, and neonatal mortality. PRDX1 is an antioxidant that regulates cell proliferation, differentiation, and apoptosis. The aim of this study is to investigate the effect of PRDX1 on the regulation of trophoblast function by affecting autophagy and oxidative stress in preeclampsia.

**Methods:**

Western blotting, RT-qPCR, and immunofluorescence were used to examine the expression of PRDX1 in placentas. PRDX1-siRNA was transfected to knockdown PRDX1 in HTR-8/SVneo cells. The biological function of HTR-8/SVneo cells was detected by wound healing, invasion, tube formation, CCK-8, EdU, flow cytometry, and TUNEL assays. Western blotting was used to detect the protein expression of cleaved-Caspase3, Bax, LC3II, Beclin1, PTEN, and p-AKT. DCFH-DA staining was used to detect ROS levels by flow cytometry.

**Results:**

PRDX1 was significantly decreased in placental trophoblasts in PE patients. Following the exposure of HTR-8/SVneo cells to H_2_O_2_, PRDX1 expression was significantly decreased, LC3II and Beclin1 expression was notably increased, and ROS level was also markedly increased. PRDX1 knockdown impaired migration, invasion, and tube-formation abilities and promoted apoptosis, which was accompanied by an increased expression of cleaved-Caspase3 and Bax. PRDX1 knockdown induced a significant decrease in LC3II and Beclin1 expression, along with an elevated p-AKT expression and a decreased PTEN expression. PRDX1 knockdown increased intracellular ROS levels, and NAC attenuated PRDX1 knockdown-induced apoptosis.

**Conclusion:**

PRDX1 regulated trophoblast function through the PTEN/AKT signaling pathway to affect cell autophagy and ROS level, which provided a potential target for the treatment of PE.

## Introduction

Preeclampsia (PE) is a relatively common and serious complication of pregnancy that is strongly associated with morbidity and mortality in pregnant women, fetuses, and newborns [1]. Hypertension and proteinuria are the main clinical manifestations of PE after the 20th week of pregnancy [2]. Pregnant women with PE have a higher risk of placental abruption, fetal intrauterine growth restriction, premature delivery, and severe complications, such as cerebrovascular dysfunction, ischemic heart disease, kidney disease, and liver injury [1, 3, 4]. Although the mechanism of PE is still unclear, it may involve abnormal placental formation characterized by the disorder of spiral artery remodeling caused by trophoblast dysfunction, which decreases placental perfusion and endothelial dysfunction [5, 6]. Thus, it is necessary to further explore the mechanisms related to trophoblast dysfunction, which may provide a potential treatment strategy for PE.

Several studies have shown that oxidative stress (OS) plays a crucial role in the occurrence and development of PE [1, 7]. OS is characterized by the excessive accumulation of reactive oxygen species (ROS) caused by the imbalance between ROS production and the antioxidant defense system, which eventually leads to cell death [1, 8]. ROS plays an important role in initiating placental and endothelial dysfunction as the second messenger. Excessive ROS can cause placental dysfunction by inhibiting placental angiogenesis and inducing endothelial damage and immune dysfunction, thus leading to PE [9, 10]. As highly reactive molecules, unwanted ROS can have a devastating impact on proteins, nucleic acids, and lipids if they cannot be eradicated by the antioxidant defense system of the cells. The antioxidant system involves superoxide dismutase, catalase, and glutathione peroxide, which are enzymatic antioxidants, vitamin C, β-carotene, and glutathione, which are non-enzymatic antioxidants [11-13]. Therefore, there is a feasible potential therapeutic strategy to prevent PE by eliminating harmful ROS and inhibiting OS.

Peroxiredoxins (PRDXs) are ubiquitous cysteine-dependent peroxidases that can eliminate H_2_O_2_, lipid peroxide, and peroxynitrite to keep the balance of oxidoreductase in the cells [14, 15]. PRDXs have been reported to have a high sensitivity to the change in H_2_O_2_ concentration [16]. As ROS scavengers, PRDXs play a major role in regulating cellular function, such as proliferation and differentiation [17]. The PRDX family consists of six human subtypes, namely PRDX1-6, which exist widely in cells [18]. PRDX1 is a typical 2-cysteine peroxidase protein that is expressed and interacts with many signaling molecules in the cytoplasm [17, 19]. PRDX1 is also found to be associated with ROS-dependent signaling pathways and is considered to be a key regulator coordinating cell signal transduction [20]. In addition to the H_2_O_2_-scavenging function, PRDX1 also acts as a molecular chaperone to regulate the effect of multiple molecules [21]. PRDX1 is involved in several signaling pathways, such as p38MAPK and PI3K/AKT, through redox-specific protein–protein interactions [14, 22]. Currently, most research on PRDX1 focuses on tumors, where it regulates tumor cell proliferation and apoptosis in vitro in several ways [23, 24]. However, the role of PRDX1 in PE development remains unclear.

Autophagy is a self-degrading process used to remove harmful cell contents and injured organelles and is involved in a variety of biological processes, such as cell growth, differentiation, and senescence [25]. Autophagy responds to changes in a variety of pathological and physiological conditions, which means it has a dual effect on cells: protecting and causing death. An increasing number of studies show that autophagy plays a significant role in several pathological and physiological processes that lead to human infertility and gestational complications [26]. It has been reported that autophagy plays an essential role in protecting trophoblast cells in the hypoxic and nutrient-restricted environment of the early stages of pregnancy [27]. However, excessive autophagy of placental trophoblasts can lead to trophoblast death, which can result in placental dysplasia [28]. Therefore, there is still much controversy regarding the specific mechanism of autophagy’s role in pregnancy-associated disease, which needs to be further explored.

In the present study, we investigated the role of PRDX1 in regulating trophoblast biological function using PE placenta samples and HTR-8/SVneo cells. Clinical samples of placental tissue were used to detect differential expression of PRDX1 in PE patients. Knockdown of PRDX1 was used to reveal the role of PRDX1 in regulating trophoblast function in the PE oxidative stress HTR-8/SVneo cell model established by H_2_O_2_. NAC was used to confirm that PRDX1 regulates trophoblast function in relation to ROS. The present study shed light on the involvement of PRDX1 in regulating the biological function of trophoblast cells in PE development and may be a potential target for the prevention and treatment of PE.

## Materials and methods

### Patient and sample collection

The clinical samples were derived from the Maternal and Child Health Care Hospital of Shandong Province. Informed consent was provided by all participants for scientific research in the study. Placental samples were selected from 10 women with PE (aged 21–34 years) and 10 normotensive pregnant women at the same age as controls (normotensive pregnancies). These patients with PE had obvious symptoms of hypertension and proteinuria, as seen by the following values: systolic blood pressure, ≥ 140 mmHg; diastolic blood pressure, ≥ 90 mmHg; proteinuria, ≥ 300 mg/day (or random proteinuria ≥ 1 +). Placental tissue was taken from the maternal side of the placenta. Placental tissue was immediately rinsed with precooled PBS to remove residual blood. Part of the tissue was stored in liquid nitrogen for RNA and protein extraction, and the rest was fixed with 4% paraformaldehyde, embedded in paraffin, and sectioned for immunofluorescence staining.

All the experiments were performed in compliance with the guideline and protocol approved by the Ethics Committee of the Maternal and Child Health Care Hospital of Shandong Province.

### Cell culture and small interfering RNA (siRNA) transfection

HTR-8/SVneo cell line, which stemmed from human placental trophoblast cells, was obtained from the American Type Culture Collection. HTR-8/SVneo cells were grown in RPMI-1640 medium (Gibco; Thermo Fisher Scientific, Inc.) supplemented with 10% fetal bovine serum (Gibco; Thermo Fisher Scientific, Inc.), 1% sodium pyruvate, and 1% penicillin–streptomycin (cat. no., P1400; Solarbio) in an incubator at 37 °C with 5% CO_2_. Different concentrations (50 μM, 150 μM, 300 μM, and 500 μM) of H_2_O_2_ were used to stimulate HTR-8/SVneo cells. N-Acetyl-L-cysteine (NAC; cat. no. A7250; Sigma‑Aldrich; Merck KGaA) is an ROS inhibitor used to treat HTR-8/SVneo cells.

For the transient transfection assay, the siRNA against human PRDX1 (PRDX1-siRNA, 5’-CAUCAAGCCUGAUGUCCAA-3’) and the corresponding negative control (scramble-siRNA) were designed and synthesized by Tsingke Biotechnology. The above siRNAs were transfected into cells using the Lipofectamine RNAiMAX (cat. no. 13778150; Thermo Fisher Scientific, Inc.). The whole transfection process was conducted according to the Lipofectamine RNAiMAX reagent instructions. Subsequently, the cells were incubated with H_2_O_2_ for 24 h. The final cells were collected for reverse transcription-quantitative PCR (RT-qPCR), western blotting, immunofluorescence staining, migration, invasion, tube formation, proliferation, apoptosis, and ROS assays. The transfected cells were treated with 3 mM NAC for 90 min prior to H_2_O_2_ stimulation.

### RNA extraction and RT-qPCR

Total RNA was isolated from the placental tissue and cells using Trizol reagent (Takara Bio, Inc.). RNA (1 μg) was reverse-transcribed into cDNA using the ReverTra Ace qPCR RT kit (Toyobo Life Science). RT-qPCR was performed using SYBR Green on the LightCycler II 480 instrument (Roche Diagnostics). The expression level of the housekeeping gene GAPDH was used as a control. The expression level of mRNA was measured by calculating the 2^−ΔΔCt^ value. The primers used were as follows: PRDX1, 5′-GAAGGCATCTCGTTCAGG-3′ forward and 5′-GCACACTTCCCCATGTTT-3′ reverse; GAPDH, 5′-GTCTCCTCTGACTTCAACAGCG-3′ forward and 5′-ACCACCCTGTTGCTG TAGCCAA-3′ reverse.

### Protein isolation and western blotting

Placental tissues were ground on ice using a tissue homogenizer (Wuhan Servicebio Technology Co., Ltd.) and dissolved in RIPA lysate (cat. no. P0013B; Beyotime Institute of Biotechnology) with 1 mmol/l phenylmethylsulfonyl fluoride (cat. no. ST506; Beyotime Institute of Biotechnology). The protein of each sample was quantified using a BCA kit (cat. no. PC0020; Solarbio). Protein (30 µg) was separated by SDS-PAGE and then transferred to a PVDF membrane, followed by blocking with 5% milk for 1 h at room temperature. The blots were incubated with specific primary antibodies overnight at 4 °C. The primary antibodies were as follows: anti-PRDX1 (cat. no. 15816–1-AP; dilution, 1:2000; ProteinTech), anti-LC3A/B (cat. no. 12741; dilution, 1:500; Cell Signaling Technology, Inc.), anti-Beclin1(cat. no. 3495; dilution, 1:1000; Cell Signaling Technology, Inc.), anti-Bax (cat. no. 2772; dilution, 1:1000; Cell Signaling Technology, Inc.), anti-cleaved-Caspase3 (cat. no. 9664; dilution, 1:500; Cell Signaling Technology, Inc.), anti‑phosphorylated (p)‑AKT (cat. no. 4060; dilution, 1:1000; Cell Signaling Technology, Inc.), anti‑PTEN (cat. no. 9188; dilution, 1:1000; Cell Signaling Technology, Inc.), and anti‑β‑actin (cat. no. 66009–1-Ig; dilution, 1:2000; ProteinTech). The next day, the blots were incubated with HRP-conjugated secondary antibodies (cat. no. GB23204 or GB23301; dilution, 1:3000; Servicebio) at room temperature for 1 h. The protein bands were generated using the ECL Detection Kit (Thermo Fisher Scientific, Inc.) and semi-quantified using an Amersham Imager 600 (Amersham; Cytiva). The results were analyzed using ImageJ software (National Institutes of Health).

### Cell proliferation assay

A cell counting kit-8 (CCK-8) assay was performed to detect cell viability. The transfected cells (6 × 10^3^/well) were seeded in 96-well plates and were stimulated with H_2_O_2_ for 24 h. Then, CCK-8 (10 μl/well; Elabscience) was added to the cells for 1 h. The absorbance at 450 nm was tested using a microplate reader (BioTek Instruments, Inc.).

5-ethynyl-2′-deoxyuridine (EdU) was used to examine cell proliferation. The transfected cells (6 × 10^3^/well) were seeded in a 96-well plate and were stimulated with H_2_O_2_ for 24 h. An EdU kit (Beyotime Institute of Biotechnology) was used to measure cell proliferation in accordance with the manufacturer’s instructions. The cells were covered by DAPI solution (Boster Biological Technology) for 15 min at room temperature. The images and positive rates were achieved by ImageXpress Micro Confocal (Molecular Devices, LCC).

### Wound healing assay

The transfected cells (4 × 10^5^/well) were seeded in a 6-well plate. A wounded scrape was created using a sterile 200 μl tip crossing the cell layer. Cell debris was dislodged by rinsing with PBS, and the FBS‑free medium was added to the cells with H_2_O_2_ stimulation for 24 h. Wound closing was monitored and photographed at 0 and 24 h using a phase contrast microscope (Zeiss GmbH). The tube length was analyzed using ImageJ software.

### Cell invasion assay

Cell invasion assays were conducted in an 8-µm pore Transwell chamber (Corning, Inc.) with Matrigel® (BD Biosciences). The Transwell chamber was covered by 60 µl Matrigel diluted with RPMI-1640 medium (1:8) for 1 h at 37 °C. Next, 8 × 10^4^/well-treated cells were resuspended with 100 µl serum‑free RPMI-1640 medium and planted in the top chamber. The bottom chamber was supplemented with 600 µl RPMI-1640 medium, including 10% FBS. After 24 h, the Transwell-treated cells on the chamber’s bottom surfaces were rinsed with PBS, and 4% paraformaldehyde was used to fix these cells for 30 min. Next, 0.1% crystal violet was used to stain these cells for 30 min. The stained cells were photographed using an orthogonal microscope (Olympus Corporation) and were calculated in five random fields for quantification.

### Tube formation assay

The Matrigel was thawed at 4 °C and diluted with RPMI-1640 medium at a ratio of 1:1. Next, 60 µl of diluted Matrigel was added to each well of a 96-well plate and incubated at 37 °C for 30 min. Treated cells were then added to each well (3 × 10^4^/well) and incubated at 37 °C for 4 h. Cells were then stained with 5 μM calcein AM (Beyotime Institute of Biotechnology) at 37 °C for 30 min. The stained cells were photographed using a fluorescence inverted microscope (Olympus Corporation).

### Apoptosis assay

Flow cytometry was used for measuring cell apoptosis using the Annexin V-FITC/Propidium Iodide (PI) Apoptosis Detection Kit (BD Biosciences). The treated cells were digested, washed twice with cold PBS, and then resuspended with 1 × binding buffer to make the cell concentration amount to 1 × 10^5^/100 μl. A total of 5 μl annexin V-FITC and 5 μl PI were added to the cell suspension. Cells were mixed gently and incubated at room temperature away from the light for 15 min. Each sample was then supplemented with 400 μl 1 × binding buffer and analyzed by flow cytometry (BD Biosciences).

### Immunofluorescence staining assay

The paraffin slices were baked at 58 °C for 1 h. Following dewaxing and hydration, the slices were put into boiling citric acid buffer for antigen repair and cooled at room temperature. The permeabilization of slices was performed using 0.5% Triton X-100 for 20 min. The slices were blocked with 5% BSA for 1 h and then incubated with primary antibodies against PRDX1 (cat. no. 15816–1-AP; dilution, 1:200; Proteintech) and cytokeratin 7 (CK7; cat. no. 17513–1-AP, dilution, 1:200; Proteintech) overnight at 4 °C. The slices were then incubated with FITC-goat anti-rabbit IgG (cat. no. GB22303; dilution, 1:500; Servicebio) and CY3-goat anti-mouse IgG (cat. no. GB21301; dilution, 1:500; Servicebio) secondary antibodies for 1 h at room temperature. DAPI solution (Boster Biological Technology) was then added to the slices for 15 min at room temperature. The images were generated using an inverted fluorescence microscope (Olympus Corporation).

### TUNEL assay

A TUNEL assay was conducted according to the instructions of the One-step TUNEL In Situ Apoptosis Kit (Elabscience). Briefly, the cells were fixed with 4% formaldehyde for 20 min. The permeabilization of cells was performed using 0.2% Triton X-100 for 10 min at 37 °C. Each sample was covered with 100 μl terminal deoxynucleotidyl transferase equilibration buffer for 10 min at 37 °C. The cells were exposed to 50 μl of labeled working solution at 37 °C for 60 min in the dark. DAPI solution was added to the cells for 15 min at room temperature.

### ROS staining assay

The treated cells were digested, washed twice with PBS, and then resuspended with 1 μM 2′-7′dichlorofluorescin diacetate (DCFH-DA; cat. no. 35845; Sigma-Aldrich; Merck KGaA). Cells were mixed gently and incubated away from light for 30 min at 37 °C. Following staining, cells were washed twice with PBS and then used for subsequent assays. ROS staining was estimated and analyzed using an inverted fluorescence microscope (Olympus Corporation) and the BD FACSCalibur™ Flow Cytometer (BD Biosciences).

### Statistical analysis

All experiments were performed at least three times. All data are expressed as the mean ± standard deviation (SD) and analyzed using GraphPad Prism 5 (GraphPad Software, Inc.). The differences were calculated using a two-tailed Student’s t-test. The images were analyzed using ImageJ software. *P* < 0.05 was considered to indicate a statistically significant difference.

## Results

### PRDX1 is highly expressed in placental trophoblasts of normal pregnancies and downregulated in PE patients

**Fig. 1:**
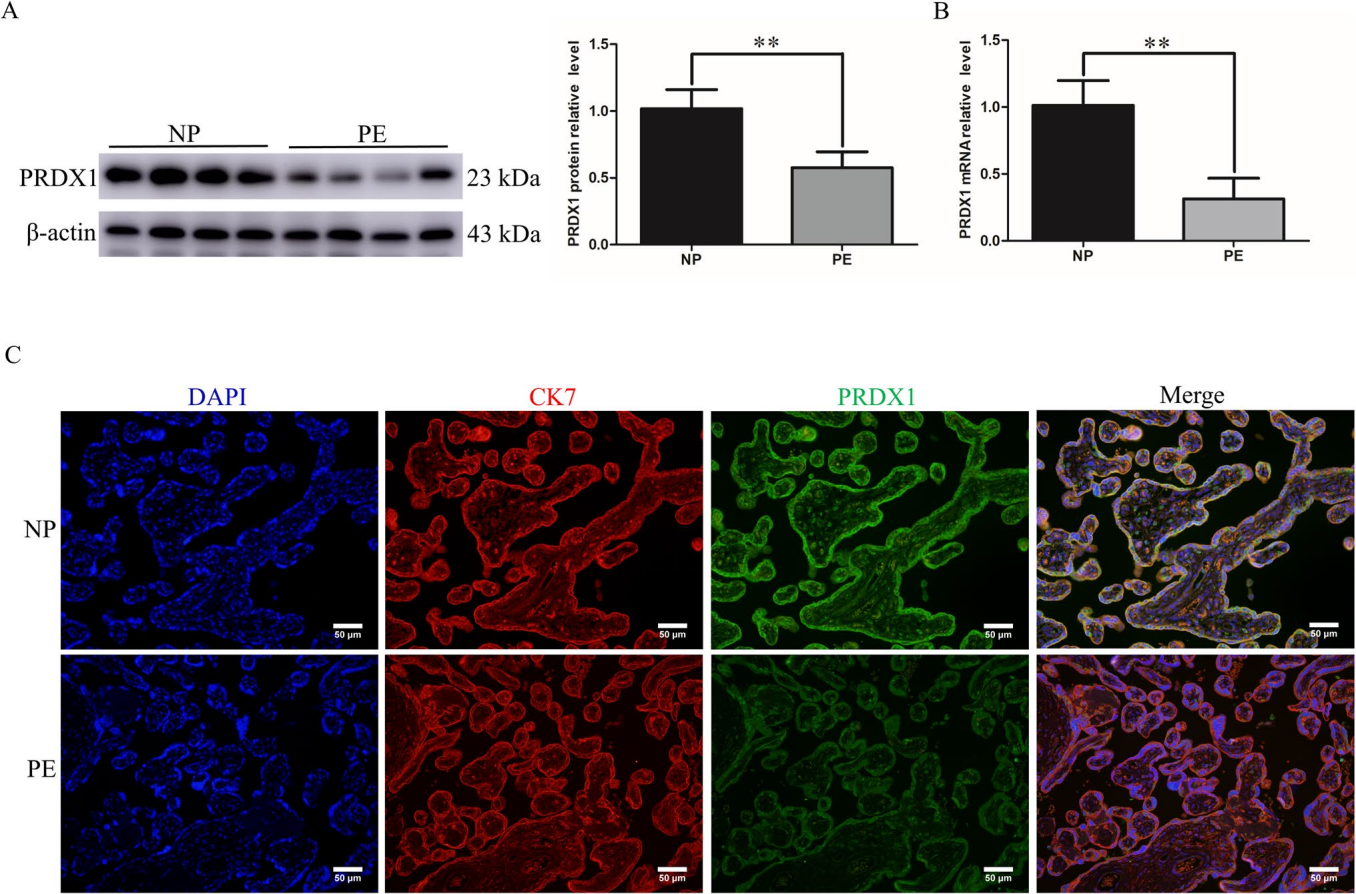
The expression of PRDX1 in human PE placenta decreases. **A** The protein expression of PRDX1 in placentas of PE and normotensive pregnancies was detected by western blotting. Protein levels were quantified by densitometry analyzing using Image J software. **B** The mRNA expression of PRDX1 in the placenta was detected using RT-qPCR. GAPDH was used as an internal parameter. **C** The expression and localization of PRDX1 were detected by immunofluorescence. Scale bars = 50 µm. All data are shown as the mean ± SD. **P < 0.01. PRDX1, peroxiredoxin 1; PE, preeclampsia; NP, normotensive pregnancies; RT-qPCR, reverse transcription-quantitative PCR

To explore the potential relationship between PRDX1 and PE, the expression of PRDX1 was detected in placental tissues from normal pregnant women and PE patients (Table 1). PRDX1 protein expression was markedly decreased in PE placentas compared with controls, as shown by western blotting analysis (Fig. 1A). The mRNA expression of PRDX1 in PE placentas was significantly lower than that of the control group, as shown by RT-qPCR (Fig. 1B). To further confirm the localization and expression of PRDX1 in PE placentas, marker of placental trophoblast cells CK7 [29] and PRDX1 double immunofluorescent staining were performed. The results showed that PRDX1 was localized in the trophoblast of the placental tissue, and the staining intensity of PRDX1 was visibly weaker than that of the control group (Fig. 1C). These results indicate that the downregulation of PRDX1 expression is associated with PE.

**Table 1:** Demographic and clinical characteristics of groups

Patient characteristics	Normotensive pregnant (*n* = 10)	Preeclampsia (*n* = 10)	*P*-value
Age (years)	26.10 ± 3.48	28.80 ± 3.77	0.1131
Gestational age (weeks)	40.03 ± 0.51	38.73 ± 1.19	0.0051
Body mass index (kg/m^2^)	27.50 ± 2.00	27.38 ± 2.68	0.9183
Systolic blood pressure (mm/Hg)	117.60 ± 4.14	150.70 ± 8.39	< 0.0001
Diastolic blood pressure (mm/Hg)	72.00 ± 4.29	93.60 ± 3.31	< 0.0001
Neonatal birth weight (g)	3305.00 ± 399.28	2979.00 ± 378.67	0.0773
Proteinuria	Negative	Positive	
Smoking status	No	No	
Abnormal fetus	No	No	
Fetal growth restriction	No	No

### HTR-8/SVneo cells treated with H_2_O_2_ results in the decrease of PRDX1 and increase of autophagy and ROS

**Fig. 2:**
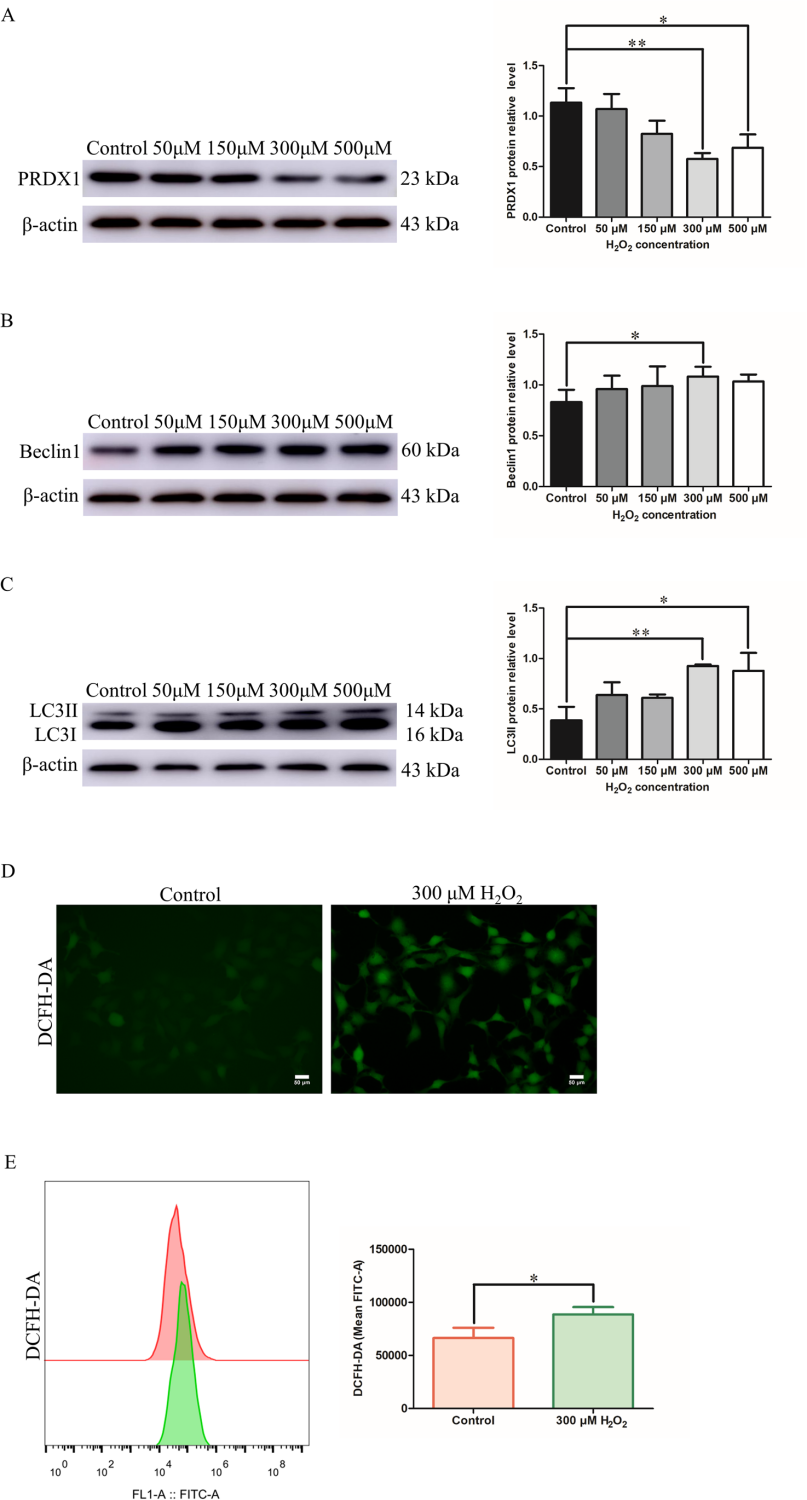
H_2_O_2_-treated HTR-8/SVneo cells resulted in the decrease of PRDX1 expression and increase of autophagy and ROS. **A**–**C** The expression of PRDX1, as well as that of autophagy-related proteins LC3II and Beclin 1, was measured by western blotting after stimulating HTR-8/SVneo cells with different concentrations of H_2_O_2_ (including 50, 150, 300, and 500 μM for 24 h). **D**, **E** The intracellular ROS level was measured by immunofluorescence and flow cytometry. Scale bars = 50 µm. All data are shown as the mean ± SD. *P < 0.05; **P < 0.01. PRDX1, peroxiredoxin 1; ROS, reactive oxygen species

H_2_O_2_ was used to imitate the OS environment of PE and construct a PE trophoblast cell model in vitro. Different concentrations of H_2_O_2_ were selected to stimulate HTR-8/SVneo cells, and then detected the changes in PRDX1 expression. Western blotting results showed that the expression of PRDX1 decreased gradually with increasing H_2_O_2_ concentrations compared with the control group (Fig. 2A). In particular, at a concentration of 300 μM, the expression of PRDX1 was the most significantly downregulated (Fig. 2A). In addition, western blotting was used to detect the expression of autophagy-related proteins, LC3II and Beclin1, in HTR-8/SVneo cells stimulated with different H_2_O_2_ concentrations. As shown, the protein expression of LC3II and Beclin1 was distinctly elevated at a concentration of 300 μM of H_2_O_2_ compared with the control group (Fig. 2B and C). In addition, the expression of ROS was also significantly elevated in H_2_O_2_-treated HTR-8/SVneo cells (Fig. 2D and E). These results demonstrate that PRDX1 may affect trophoblast autophagy and ROS in different H_2_O_2_ concentration.

### PRDX1 knockdown aggravates the inhibitory effect of H_2_O_2_ on the migration, invasion, and tube-forming capacity of HTR-8/SVneo cells

**Fig. 3:**
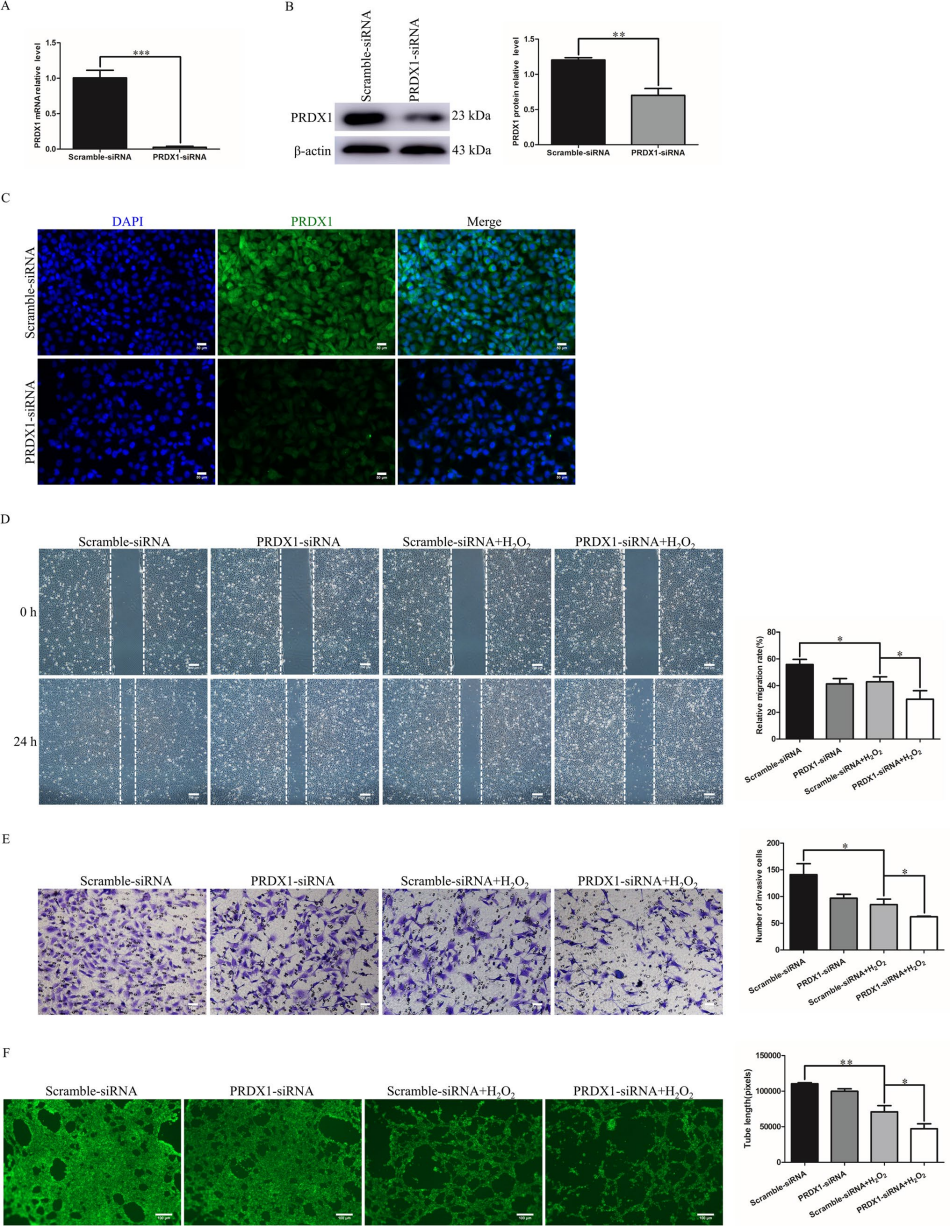
PRDX1 knockdown aggravated the inhibitory effect of H_2_O_2_ on the migration, invasion and tube-formation capacity of HTR-8/SVneo cells. **A**–**C** RT-qPCR, western blotting and immunofluorescence were used to analyze the expression of PRDX1 after 48 h of PRDX1-siRNA transfection. Scale bars = 50 µm. **D** Wound healing assays were performed to detect the effect of PRDX1 knockdown on the migration ability of HTR-8/SVneo cells treated with H_2_O_2_. Scale bars = 200 µm. **E** Transwell assays were used to examine the effect of PRDX1 knockdown on the invasive ability of H_2_O_2_-treated HTR-8/SVneo cells. Scale bars = 20 µm. **F** Tube-formation assays were used to explore the effect of PRDX1 knockdown on the tube-forming ability of HTR-8/SVneo cells treated with H_2_O_2_. Scale bars = 100 µm. All data are shown as the mean ± SD. *P < 0.05; **P < 0.01; ***P < 0.001. PRDX1, peroxiredoxin 1; RT-qPCR, reverse transcription-quantitative PCR

Given that trophoblast dysfunction is intrinsically linked to PE development, the effect of PRDX1 knockdown on the biological function of H_2_O_2_-treated HTR-8/SVneo cells was explored in vitro. HTR-8/SVneo cells were transfected with PRDX1-siRNA, and the transfection efficiency was detected by RT-qPCR and western blotting (Fig. 3A and B). The immunofluorescence results showed that PRDX1 was localized in the cytoplasm, and PRDX1 expression was significantly downregulated in PRDX1-siRNA cells compared with that in scramble-siRNA cells (Fig. 3C). To explore the role of PRDX1 in OS in trophoblasts, wound healing, Transwell, and tube formation assays were performed. The migration ability of the PRDX1-siRNA group was evidently slower than that of the scramble-siRNA group in H_2_O_2_-stimulated cells (Fig. 3D). Compared with the scramble-siRNA group, the invasive ability of the PRDX1-siRNA group was significantly decreased in H_2_O_2_-stimulated cells (Fig. 3E). The tube-formation assay showed that PRDX1 knockdown reduced total tube length, as compared with the scramble-siRNA group in H_2_O_2_-stimulated cells (Fig. 3F). The above results indicate that PRDX1 can regulate the biological function of trophoblast cells.

### PRDX1 knockdown inhibits H_2_O_2_-stimulated HTR-8/SVneo cell proliferation and induces their apoptosis

**Fig. 4:**
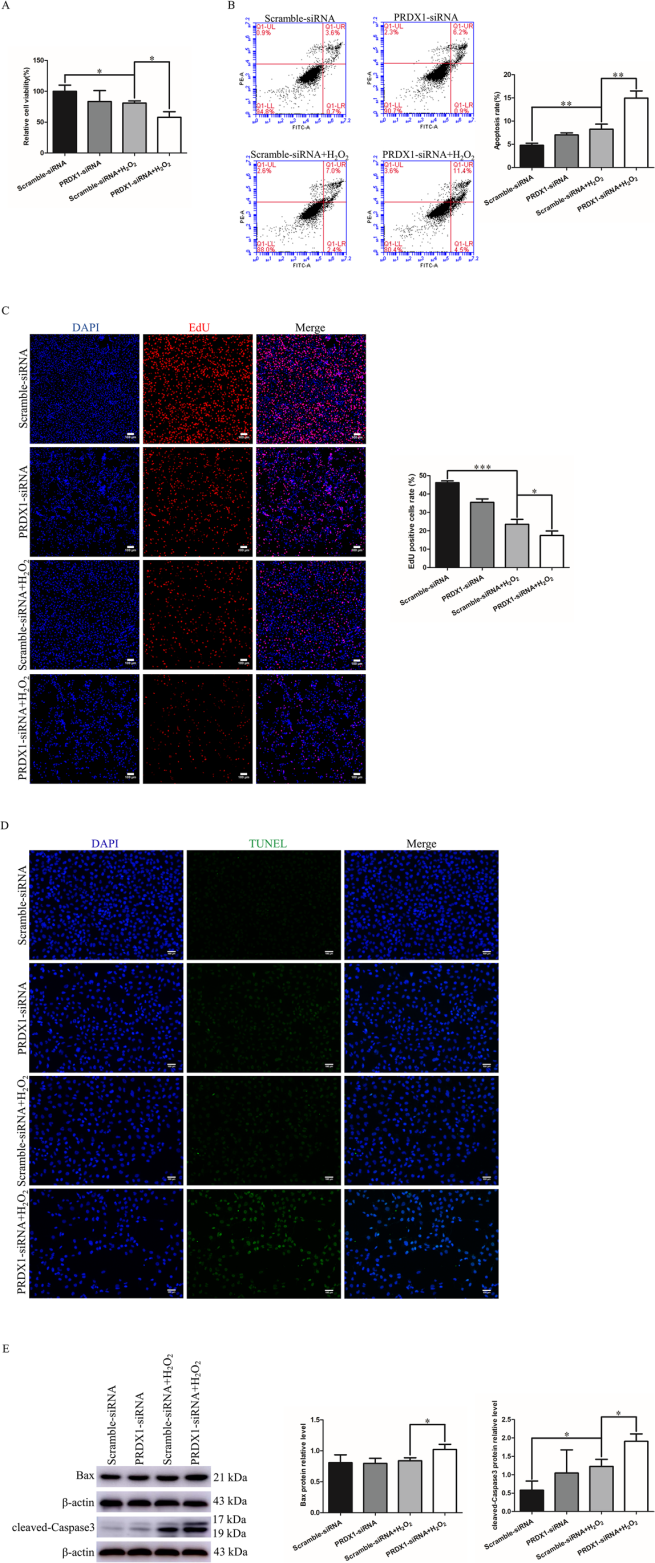
PRDX1 knockdown has an inhibitory proliferative and pro-apoptotic effect on HTR-8/SVneo cells exposed to H_2_O_2_. **A** CCK-8 assays were used to detect cell proliferation in HTR-8/SVneo cells knocked down by PRDX1 following exposure to H_2_O_2_ stimulation. **B** Flow cytometry was used to detect apoptosis in HTR-8/SVneo cells knocked down by PRDX1 following exposure to H_2_O_2_ stimulation. **C** EdU assays were used to detect cell proliferation in HTR-8/SVneo cells knocked down by PRDX1 following exposure to H_2_O_2_ stimulation. Scale bars = 100 µm. **D** TUNEL assays were performed to evaluate apoptosis in HTR-8/SVneo cells knocked down by PRDX1 following exposure to H_2_O_2_ stimulation. Scale bars = 100 µm. **E** Western blotting assays were performed to detect apoptotic protein cleaved-Caspase3 and Bax expression following PRDX1 knockdown in HTR-8/SVneo cells exposed to H_2_O_2_. All data are shown as the mean ± SD. *P < 0.05; **P < 0.01; ***P < 0.001. PRDX1, peroxiredoxin 1; CCK-8, cell counting kit-8; EdU, 5-ethynyl-2’-deoxyuridine

PRDX1-siRNA was transfected into cells to explore the effect of PRDX1 knockdown on trophoblast proliferation and apoptosis. CCK-8 assay results showed that PRDX1 knockdown aggravated cell damage in H_2_O_2_-stimulated HTR-8/SVneo cells (Fig. 4A). Annexin V-FITC/PI double staining was used to measure HTR-8/SVneo cell apoptosis through flow cytometry. The results showed that PRDX1 knockdown increased the apoptosis of HTR-8/SVneo cells after H_2_O_2_ stimulation by flow cytometry (Fig. 4B). EdU assay demonstrated that PRDX1 knockdown reduced the proportion of EdU-labeled positive cells (Fig. 4C). TUNEL staining assays showed that PRDX1 knockdown resulted in an increase in apoptosis (Fig. 4D). Western blotting results showed that PRDX1 knockdown led to a distinct elevation in the expression of apoptosis markers cleaved-Caspase3 and Bax protein following H_2_O_2_ exposure (Fig. 4E). In conclusion, these results suggest that PRDX1 knockdown leads to the inhibition of proliferation and promotion of apoptosis in HTR-8/SVneo cells following H_2_O_2_ treatment.

### PRDX1 knockdown attenuates H_2_O_2_-induced autophagy by regulating the PTEN/AKT pathway

**Fig. 5:**
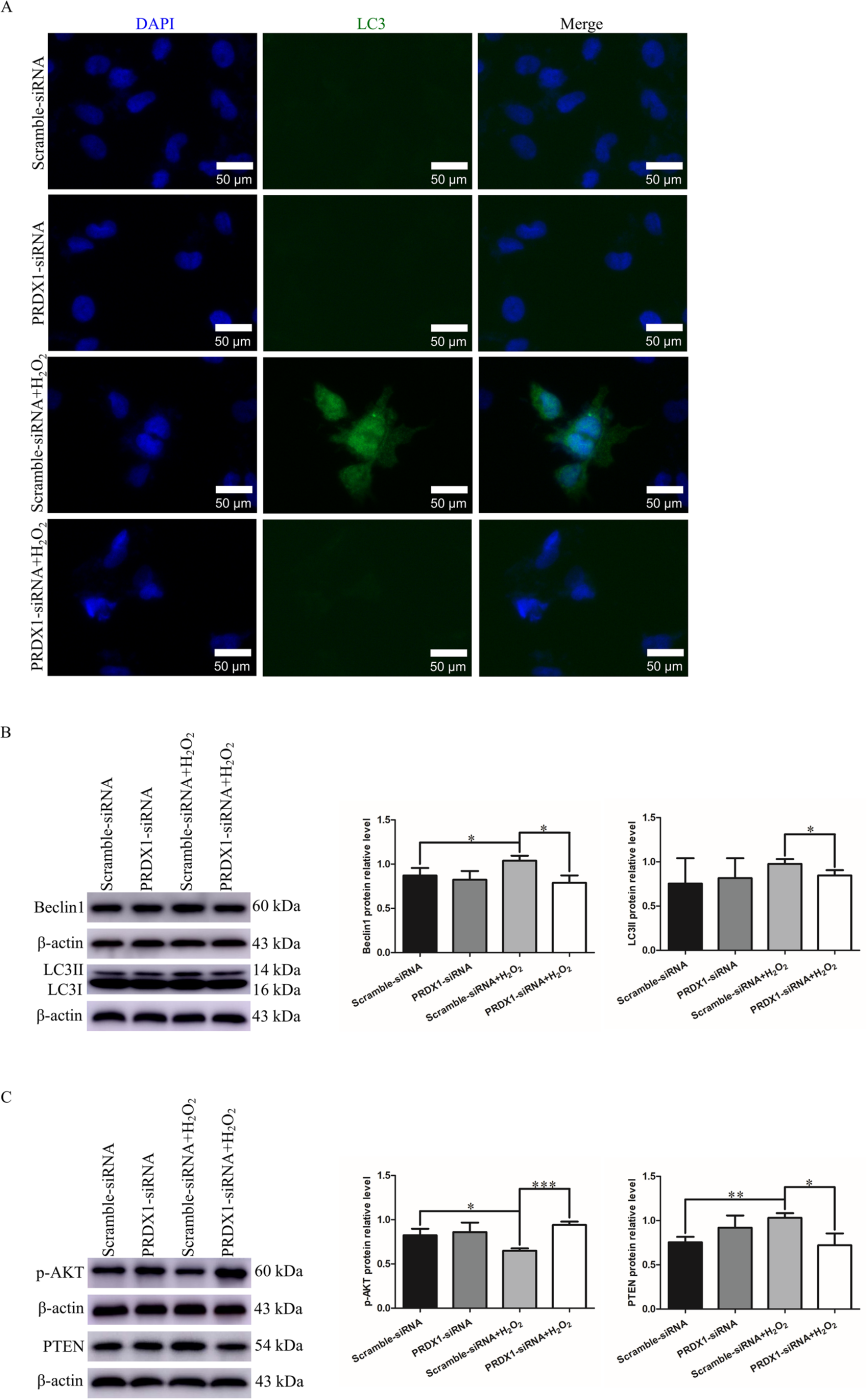
PRDX1 knockdown attenuates H_2_O_2_-induced autophagy by regulating the PTEN/AKT pathway. **A** Immunofluorescence assays were performed to detect the effect of PRDX1 knockdown on H_2_O_2_-induced autophagy in HTR-8/SVneo cells. Scale bars = 50 µm. **B** Western blotting was performed to examine the effect of PRDX1 knockdown on the expression of H_2_O_2_-induced autophagy-related proteins LC3II and Beclin1 in HTR-8/SVneo cells. **C** Western blotting was performed to examine the protein expression of p-AKT and PTEN in HTR-8/SVneo cells using different experimental settings. All data are shown as the mean ± SD. *P < 0.05; **P < 0.01; ***P < 0.001. PRDX1, peroxiredoxin 1

To further explore the mechanism of PRDX1 in regulating the proliferation and apoptosis of trophoblasts, the expression of autophagy-related proteins and the potentially related signaling pathway were examined. Immunofluorescence staining showed that the autophagy marker LC3 was significantly elevated in HTR-8/SVneo cells treated with H_2_O_2_, while it was not detected in PRDX1 knockdown and H_2_O_2_-treated cells (Fig. 5A). Western blotting showed that following H_2_O_2_ exposure, the expression of autophagy-related proteins, including LC3II and Beclin1, were increased in the scramble-siRNA group, while PRDX1 knockdown inhibited their increase (Fig. 5B). In H_2_O_2_-treated HTR-8/SVneo cells, phosphorylated (p)-AKT protein expression was significantly reduced and PTEN protein expression was markedly elevated, while PRDX1 knockdown reversed this change, as shown by western blotting (Fig. 5C). These results suggest that PRDX1 regulates trophoblast autophagy via the PTEN/AKT pathway.

### PRDX1 knockdown enhances H_2_O_2_-induced ROS levels in HTR-8/SVneo cells, and NAC attenuates PRDX1 knockdown-induced apoptosis

**Fig. 6:**
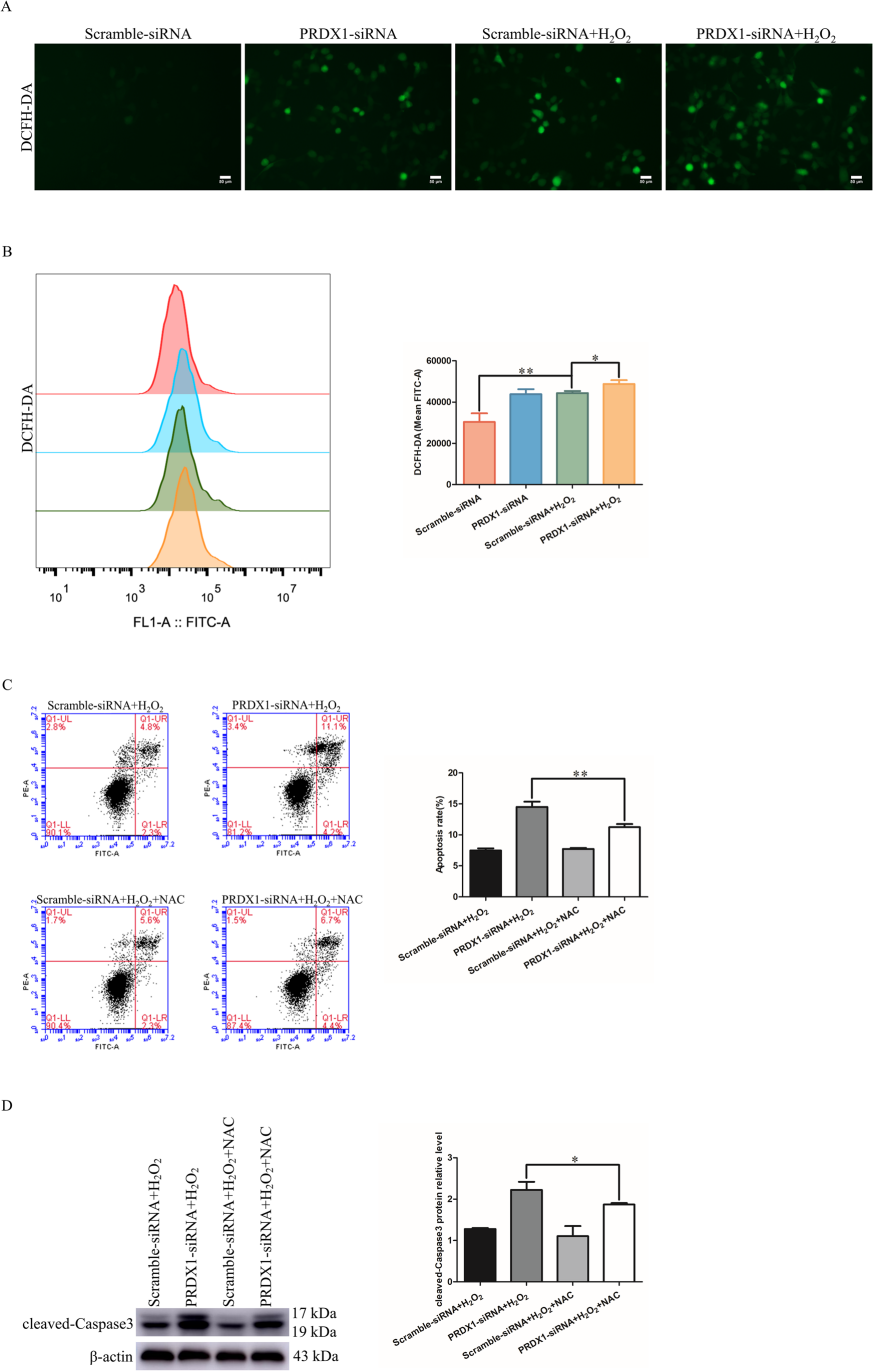
PRDX1 knockdown enhances H_2_O_2_-induced ROS levels in HTR-8/SVneo cells, and NAC, as an inhibitor of ROS, attenuates PRDX1 knockdown-induced apoptosis. **A**, **B** DCFH-DA staining and flow cytometry were performed to detect the effect of PRDX1 knockdown on H_2_O_2_-induced ROS level in HTR-8/SVneo cells. Scale bars = 50 µm. **C** Flow cytometry was used to detect apoptosis in HTR-8/SVneo cells using several experimental settings. **D** Western blotting was performed to examine the protein expression of cleaved-Caspase3 in HTR-8/SVneo cells using several experimental settings. All data are shown as the mean ± SD. *P < 0.05, **P < 0.01. PRDX1, peroxiredoxin 1; NAC, N-acetyl-L-cysteine; ROS, reactive oxygen species

To explore the role of PRDX1 and ROS in PE, the ROS inhibitor NAC was used to detect apoptosis in HTR-8/SVneo cells. DCFH-DA staining was performed to assess the expression levels of intracellular ROS. The results showed that the PRDX1 knockdown resulted in a significant increase in ROS level following H_2_O_2_ exposure in HTR-8/SVneo cells, as shown by fluorescent staining and flow cytometry (Fig. 6A and B). NAC attenuated PRDX1 knockdown-induced apoptosis following H_2_O_2_ stimulation through flow cytometry (Fig. 6C). PRDX1 knockdown led to a significant increase in cleaved-Caspase3 protein expression, while NAC reversed this effect (Fig. 6D). It is speculated that the regulation of PRDX1 on proliferation and apoptosis of trophoblasts is closely linked to ROS.

## Discussion

The placenta is a crucial organ involved in the exchange of gases, nutrients, and waste products between the mother and the fetus and maintains homeostasis in the mother-fetus interface [30]. Placental hypoxia during pregnancy is connected to excessive ROS production, leading to OS, which in turn causes endothelial cell injury and systemic vessel constriction [31]. OS is thought to be an imbalance between pro- and anti-oxidant capacity [32]. PE is caused by hypoxia-induced placental dysfunction during pregnancy. The increase in OS in the placenta is closely related to the onset of PE [33]. As a complication of pregnancy, PE is characterized by an injured fetal trophoblastic infiltrate, which leads to the abnormal remodeling of the spiral arteries, resulting in the reduction of blood flow between mother and fetus [34]. There is no accepted method to prevent PE. Therefore, it is of great significance to clarify the mechanism of placental trophoblast dysfunction for the prevention and treatment of PE.

ROS is a double-edged sword. When the level of ROS is low, it acts as a signal molecule to regulate a wide range of cellular processes. On the contrary, excessive accumulation of ROS damages cellular macromolecules, thus causing damage to cellular functions [35]. The excessive accumulation of trophoblast ROS induces OS, which is a crucial factor in the development of PE [36]. HTR-8/SVneo cell line was generated from early gestational extravillous trophoblasts infected with SV40 Large T antigen, which is often chosen for research on the pathogenesis of placental diseases [9]. H_2_O_2_ was selected for modeling OS in vitro [37].

In normal physiological conditions, antioxidant enzymes containing catalase, glutathione peroxidase, and peroxiredoxins can convert ROS to H_2_O to prevent excessive ROS production [11, 35]. PRDX1 is a member of the peroxiredoxin family and has been proven to be a multifunctional molecule for regulating cell growth, differentiation and apoptosis [23]. It has been reported that PRDX1 can reduce ROS and inhibit tumor cell apoptosis [20]. Recent studies have shown that PRDX1 prevents the damage of hydrogen peroxide and peroxynitrite to pancreatic β cells [38]. However, the role of PRDX1 in regulating trophoblast function is virtually unknown. The present study found that PRDX1 knockdown elevated ROS levels in HTR-8/SVneo cells with and without H_2_O_2_ stimulation. PRDX1 knockdown led to HTR-8/SVneo cell dysfunction, including migration and invasion inhibition and tube-forming capacity impairment. Moreover, PRDX1 knockdown inhibited HTR-8/SVneo cell proliferation and promoted apoptosis upon exposure to H_2_O_2_. PRDX1 expression inhibition resulted in a significant increase in the expression levels of the apoptosis-related proteins, cleaved-Caspase3 and Bax, following H_2_O_2_ treatment. In addition, NAC as a ROS inhibitor was able to attenuate PRDX1 knockdown-induced apoptosis following H_2_O_2_ treatment. Thus, these results indicate that PRDX1 affects trophoblast function by regulating ROS in PE.

Autophagy is a catabolic process that maintains cell growth and differentiation, as well as homeostasis under harmful conditions such as nutrient deprivation and hypoxia, by removing excess proteins and damaged organelles (e.g., peroxisomes and mitochondria) [39]. Autophagy has been identified as an essential mediator of pathological responses, which is related to ROS in cell signaling and cell injury [40]. ROS can directly oxidize autophagy regulatory proteins, such as SUMO-specific peptidase 3, autophagy-related 3 (ATG3), and ATG7, thus inhibiting autophagy [41]. PRDX1 is reported to inhibit cellular autophagy activation by negatively regulating ubiquitination [18]. PRDX1 can also activate autophagy and inhibit ROS accumulation and apoptosis in spiral ganglion neurons through the PTEN-AKT pathway [42]. PRDX1 deletion has an impact on biological pathways critical to the survival of pancreatic ductal adenocarcinoma cells, as well as STAT3 and autophagy [43]. In the present study, PRDX1 exerted an inhibitory effect on H_2_O_2_-induced elevated expression of autophagy-related proteins LC3II and Beclin1. In addition, PRDX1 promoted the proliferation and inhibited the apoptosis of HTR-8/SVneo cells by inhibiting the accumulation of ROS. It is speculated that PRDX1 affects the biological function of trophoblast cells by inhibiting autophagy activation through regulating ROS production.

PRDX1 participates in various physiological activities of different cells in its own specific ways, which in turn regulates the biological functions of cells. It has been reported that PRDX1 overexpression can attenuate OS and cardiomyocyte apoptosis through the ASK1/p38 pathway, thereby ameliorating doxorubicin-induced cardiotoxicity [44]. PRDX1 has been shown to repress nasopharyngeal carcinoma cell proliferation, migration, invasion, and epithelial-mesenchymal transition in vitro by inactivating the PI3K/AKT/TRAF1 pathway [45]. PRDX1 knockdown leads to the inhibition of proliferation and increase of apoptosis in non-small cell lung cancer cells through the Wnt/β-catenin pathway [24]. PRDX1 deficiency induces an increase in the death of streptozotocin-treated pancreatic β-cells via the AKT/GSK-3β/β-catenin signaling pathway [46]. PRDX1 is involved in the inhibition of tumorigenesis by regulating the PTEN/AKT signaling pathway [47] and the activation of NF-κB and autophagy of TLR4 induction by restraining the activity of TRAF6 ubiquitin ligase [18]. PRDX1 has been reported to inhibit AKT-driven tumorigenesis and then invoke cell death by preventing the oxidative-induced inactivation of PTEN lipid phosphatase activity [20]. In the present study, PRDX1 knockdown resulted in an increased p-AKT expression and reduced PTEN expression in H_2_O_2_-treated HTR-8/SVneo cells. These results suggest that PRDX1 knockdown regulates trophoblast function might through the PTEN/AKT pathway following H_2_O_2_ treatment in vitro. However, the specific role between PRDX1 and PTEN-AKT needs to be further explored.

There are some limitations in our study, such as the type and number of PE clinical samples. In vivo animal experiments are also needed for validation in our study. We will next delve into the mechanisms of PRDX1 action in PE by increasing the type and number of samples and in vivo experiments in mice.

In conclusion, the present study demonstrated that PRDX1 expression was markedly decreased in trophoblasts in PE placenta. These results provided evidence that PRDX1 knockdown impaired the biological functions of migration, invasion, and tube formation in HTR-8/SVneo cells exposed to H_2_O_2_. Furthermore, PRDX1 knockdown inhibited proliferation and promoted apoptosis through autophagy inhibition and ROS accumulation pathways in HTR-8/SVneo cells exposed to H_2_O_2_. Mechanistically, PRDX1 knockdown regulated trophoblast function in HTR-8/SVneo cells following H_2_O_2_ stimulation via the PTEN/AKT pathway. These findings support the role of PRDX1 as an emerging and essential factor in the regulation of the biological function of trophoblasts and may provide new clues for understanding the molecular mechanisms of PE.

## Data Availability

All data of this study are available from the corresponding author on reasonable request.
